# Recurrent Ovarian Torsion in a Premenarchal Girl Managed With Laparoscopic Round Ligament Oophoropexy: A Case Report

**DOI:** 10.7759/cureus.110206

**Published:** 2026-06-03

**Authors:** Elisavet Kanna, Zoi Lamprinou, Despina Panayiotou, Eleni Batsari, Ioannis Skondras

**Affiliations:** 1 2nd Pediatric Surgery Department, Panagiotis and Aglaia Kyriakou Children's Hospital, Athens, GRC; 2 Otolaryngology Department, Panagiotis and Aglaia Kyriakou Children's Hospital, Athens, GRC; 3 Nursing Department, National and Kapodistrian University of Athens, Athens, GRC

**Keywords:** adnexal torsion, laparoscopy, oophoropexy, ovarian preservation, ovarian torsion, pediatric, recurrent ovarian torsion, round ligament fixation

## Abstract

Ovarian torsion is an uncommon but important cause of acute abdominal pain in the pediatric population and may occur even in the absence of an underlying ovarian mass. Recurrent torsion is rare and poses a significant therapeutic challenge, with no clear guidelines regarding optimal management or fixation technique. We report the case of a 12-year-old premenarchal girl with recurrent torsion of the left ovary, experiencing four episodes over a four-month period despite repeated laparoscopic detorsion and preservation of ovarian viability. During the most recent episode, laparoscopy revealed a left ovary twisted four times around its vascular pedicle. Following detorsion, oophoropexy was performed, and the ovary was fixed to the round ligament of the uterus with careful identification of the ureter. The postoperative course was uneventful. This case highlights the importance of early recognition, ovarian preservation, and individualized surgical decision-making while also emphasizing the need to consider potential implications for future fertility when selecting the appropriate surgical technique.

## Introduction

Ovarian torsion is an uncommon but important cause of acute abdominal pain in the pediatric population, accounting for approximately 15-20% of all cases [1]. Although it may occur at any age, it is most frequently observed in neonates and postmenarchal adolescents. Delayed diagnosis can result in ovarian ischemia and loss of reproductive potential, emphasizing the need for prompt recognition and surgical intervention [1,2].

Ovarian torsion results from partial or complete rotation of the adnexa around its vascular pedicle, leading initially to venous and lymphatic obstruction, followed by arterial compromise and ovarian ischemia if left untreated [1].

Clinically, ovarian torsion usually presents with acute lower abdominal pain, frequently accompanied by nausea or vomiting [3]. However, the clinical presentation may be nonspecific in children, making the diagnosis challenging and potentially delaying treatment. Early recognition and prompt surgical detorsion are essential for the preservation of ovarian function.

In children, ovarian torsion often occurs in the absence of an underlying ovarian mass and is associated with increased ovarian mobility. Proposed predisposing factors include elongated utero-ovarian ligaments, congenital anatomic variations, and ovarian enlargement related to hormonal changes [1,2]. Recurrent ovarian torsion is rare and poses a therapeutic challenge. Several oophoropexy techniques have been described to prevent recurrence; however, there are no clear guidelines regarding ovarian fixation, and concerns remain regarding potential effects on future fertility [3]. While oophoropexy may reduce the risk of recurrent torsion, recurrence has still been reported [4].

Reported recurrence rates after conservative detorsion vary in the literature and appear to be higher in patients with torsion of otherwise normal adnexa, prompting the consideration of oophoropexy in selected cases [3]. Among the various fixation techniques, round ligament oophoropexy may represent a useful alternative when plication of the utero-ovarian ligament or fixation to the pelvic sidewall is technically challenging or not feasible [1,4].

We present the case of a 12-year-old girl with multiple episodes of ovarian torsion successfully managed with oophoropexy, specifically by fixation of the ovary to the round ligament of the uterus, aiming to highlight the diagnostic challenges, therapeutic considerations, and current evidence regarding ovarian fixation in the pediatric population.

## Case presentation

A 12-year-old premenarchal girl presented to the emergency department with acute-onset lower abdominal pain. Her medical history was significant for four episodes of left ovarian torsion occurring at monthly intervals over a four-month period. The previous episodes had been managed at other institutions, and detailed information regarding the decision-making process at those admissions was unavailable. In each episode, the diagnosis was suggested by ultrasound findings and confirmed intraoperatively during laparoscopic detorsion.

**Figure 1:**
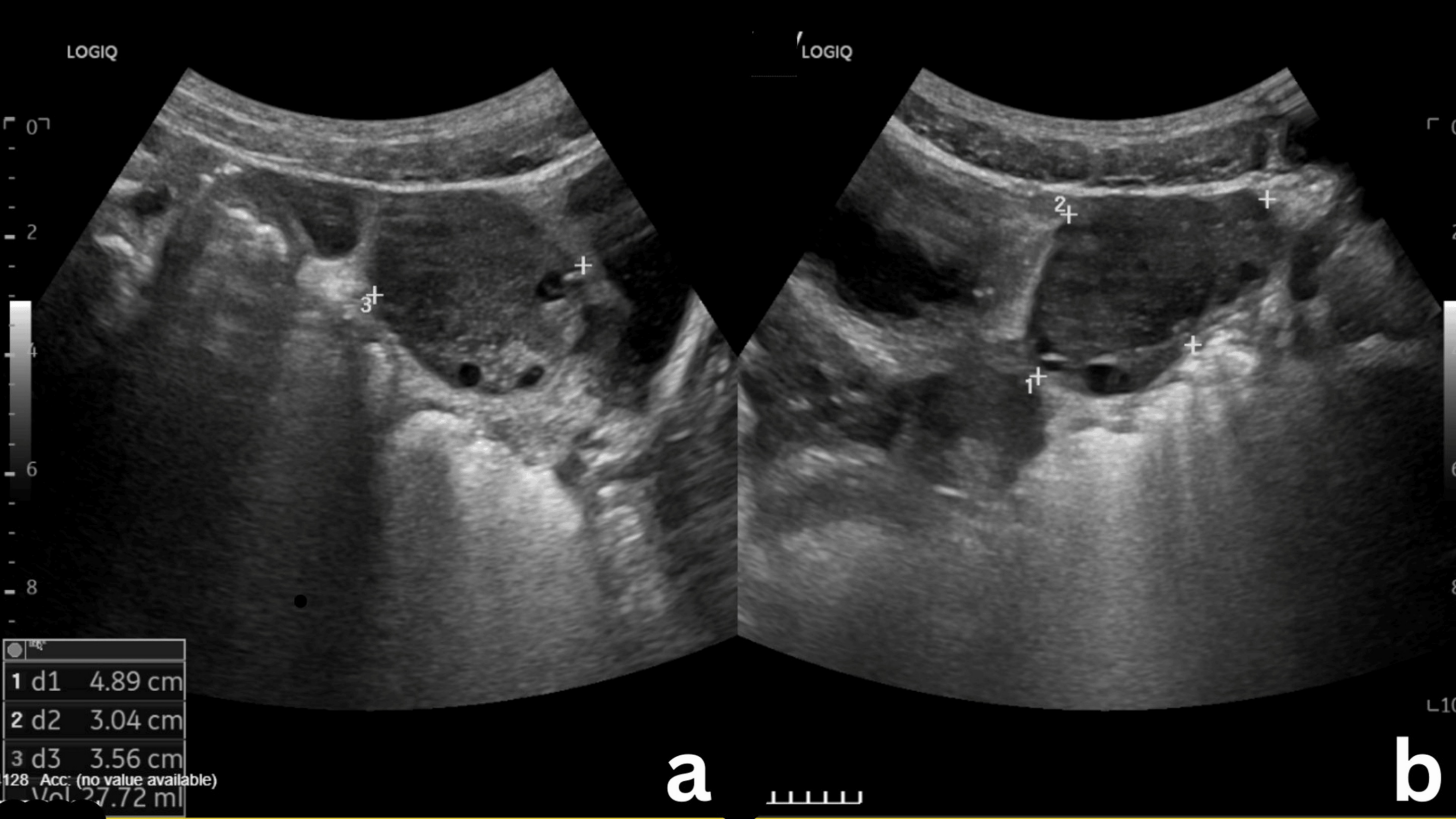
Ultrasound findings of the left ovary (a) Enlarged ovary with stromal edema and peripheral displacement of follicles. (b) Ultrasound measurements demonstrating increased ovarian dimensions and an ovarian volume of 26.72 mL.

During each episode, the patient presented with severe lower abdominal pain requiring hospital admission and surgical evaluation. Transabdominal ultrasound examination using a LOGIQ ultrasound system (GE Healthcare, Chicago, Illinois, United States) demonstrated an enlarged left ovary with stromal edema and peripheral follicles, findings consistent with ovarian torsion (Figure 1). Laparoscopic detorsion of the left ovary was performed after each episode in order to preserve ovarian viability. Despite restoration of ovarian blood flow following each procedure, the patient continued to experience recurrent torsion.

**Figure 2:**
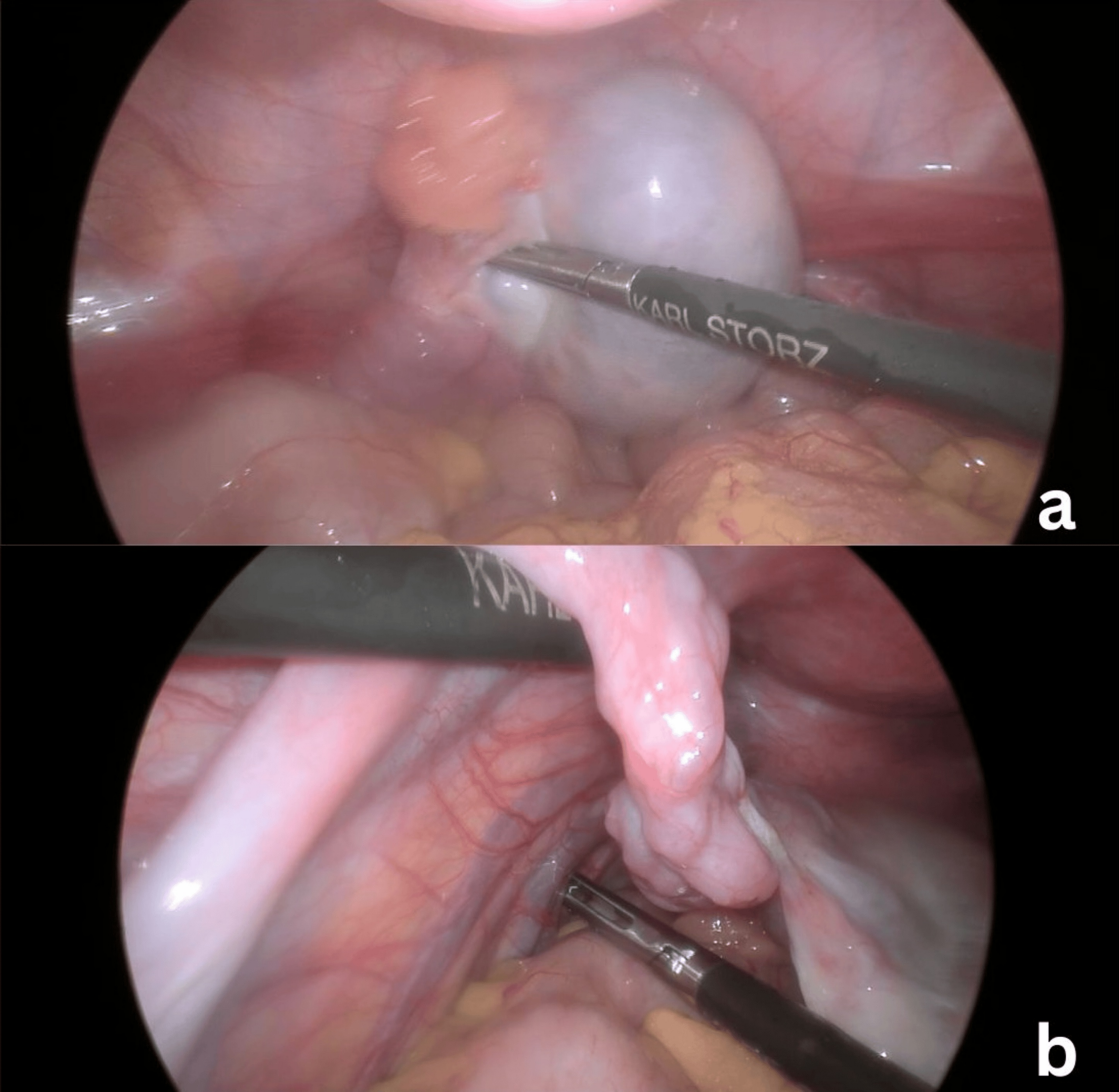
Intraoperative findings (a) Left ovarian torsion with twisting of the vascular pedicle and ovarian enlargement. (b) Markedly dilated pelvic venous plexus, which made fixation to the pelvic sidewall technically unsafe.

Given the repeated episodes and the risk of further ovarian compromise, surgical ovarian fixation was considered. During the most recent episode, laparoscopy confirmed torsion of the left ovary, twisted four times around its vascular pedicle (Figure 2). After detorsion and assessment of ovarian viability, an oophoropexy was performed to prevent further recurrence. The procedure was carried out using standard laparoscopic instruments (Karl Storz, Tuttlingen, Germany).

Fixation of the ovary to the pelvic sidewall was considered; however, this approach was deemed technically difficult because of the presence of a markedly dilated pelvic venous plexus, which increased the risk of vascular injury. In addition, plication of the utero-ovarian ligament was not feasible because of the significantly enlarged ovary.

**Figure 3:**
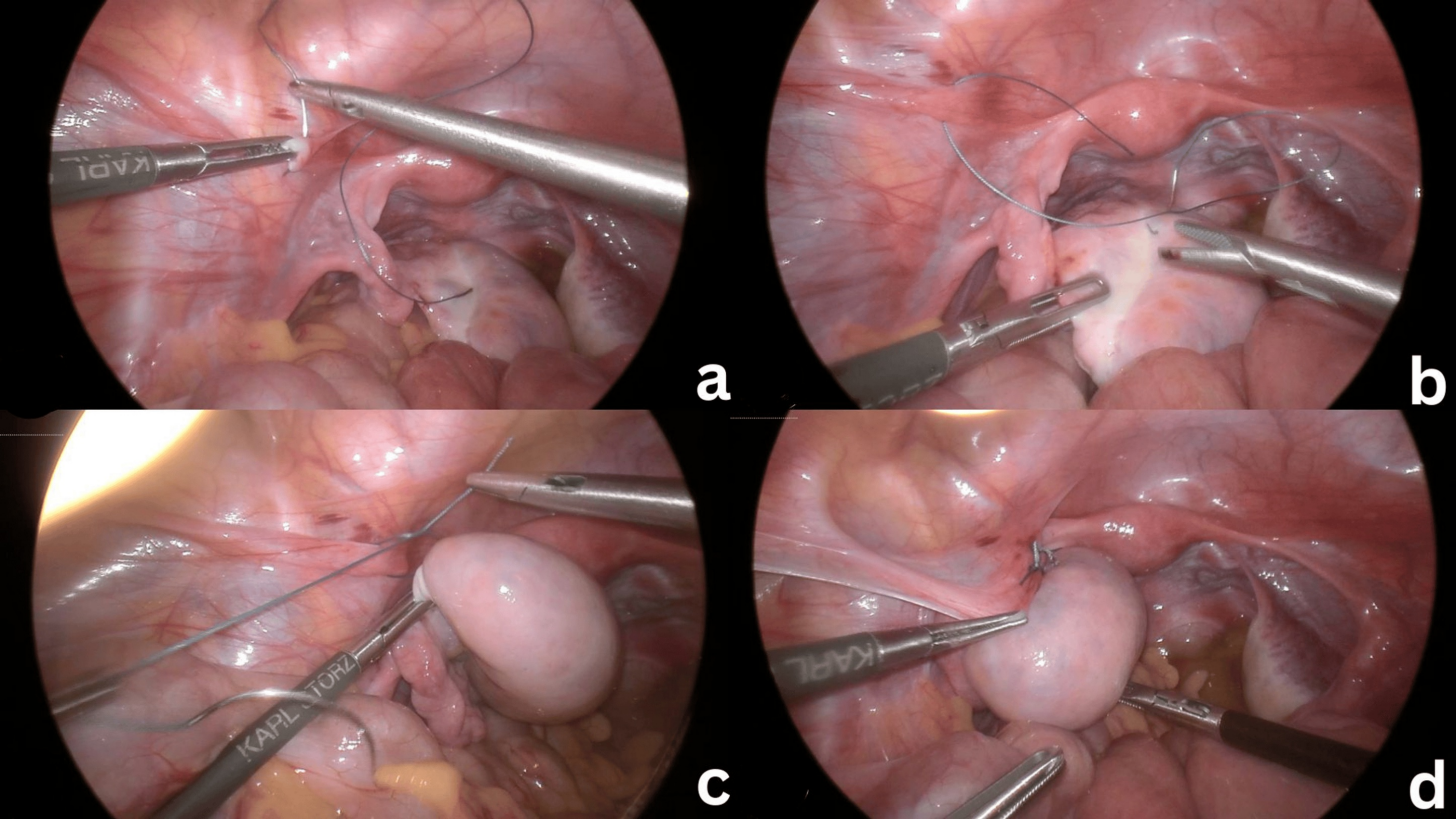
Laparoscopic oophoropexy to the round ligament of the uterus (a) Placement of suture on the round ligament. (b) Placement of suture on the ovary. (c) Knot tying and approximation of the ovary to the round ligament. (d) Ovary after fixation with two sutures to the round ligament of the uterus.

**Figure 4:**
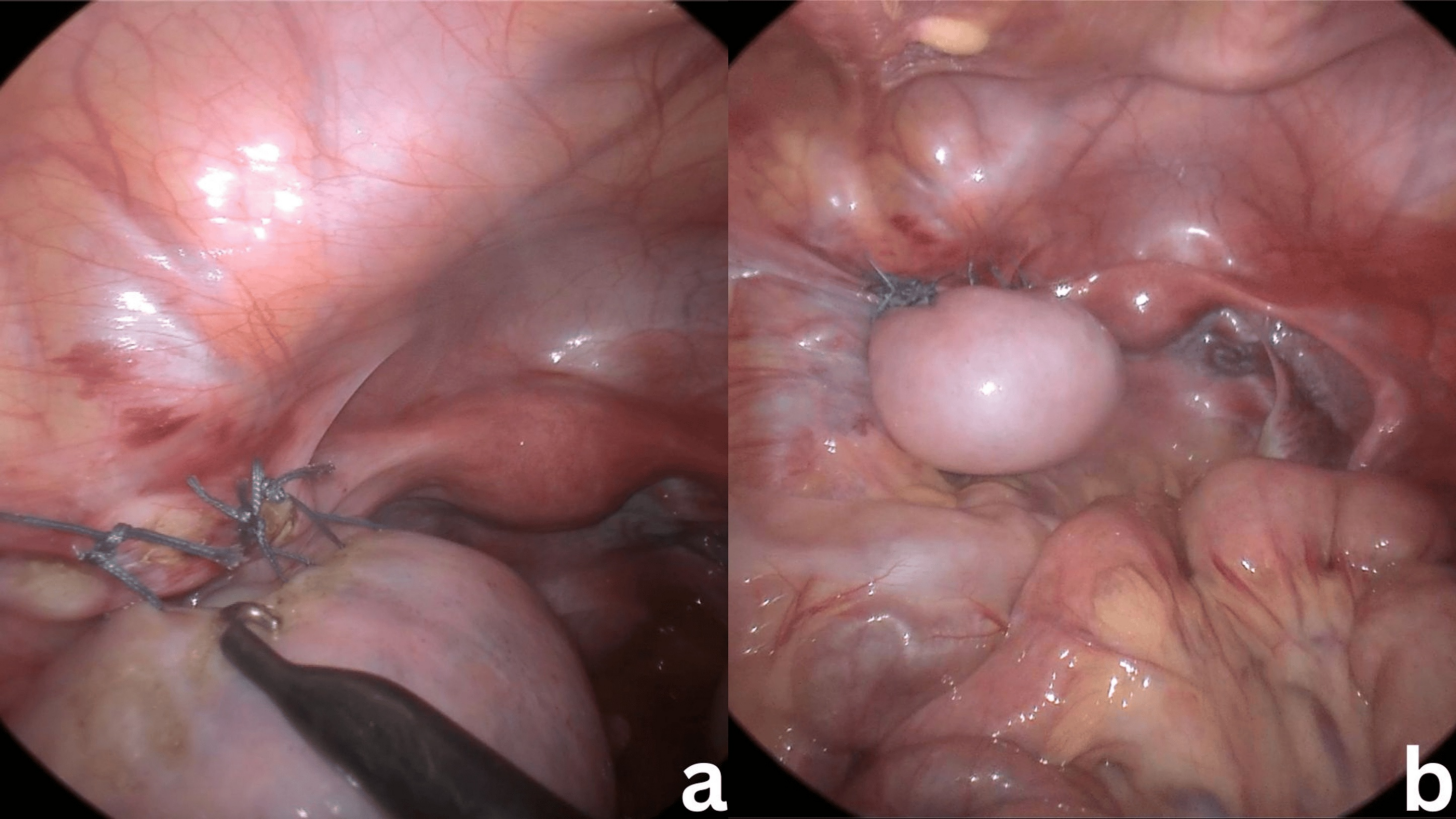
Final steps of laparoscopic oophoropexy (a) Creation of a raw surface on the round ligament to facilitate the secure fixation of the ovary. (b) Final intraoperative appearance of the left ovary in stable position after fixation to the round ligament of the uterus.

A 2-0 Ethibond suture (Ethicon, Somerville, New Jersey, United States) on a 16-mm needle was introduced via the umbilical trocar, with slight curvature of the needle to allow easier intracorporeal manipulation. Therefore, the left ovary was surgically fixed to the round ligament of the uterus using a 2-0 Ethibond suture mounted on a 16-mm needle. Multiple passes secured with sliding knots were performed to achieve stable and tension-free fixation after careful identification and protection of the ureter, aiming to reduce the risk of recurrent torsion (Figures 3-4).

The procedure was completed laparoscopically without complications. The ovary appeared viable following detorsion, and adequate blood flow was restored. The patient had an uneventful postoperative course and was discharged in stable condition. Follow-up evaluation showed no immediate postoperative complications.

**Figure 5:**
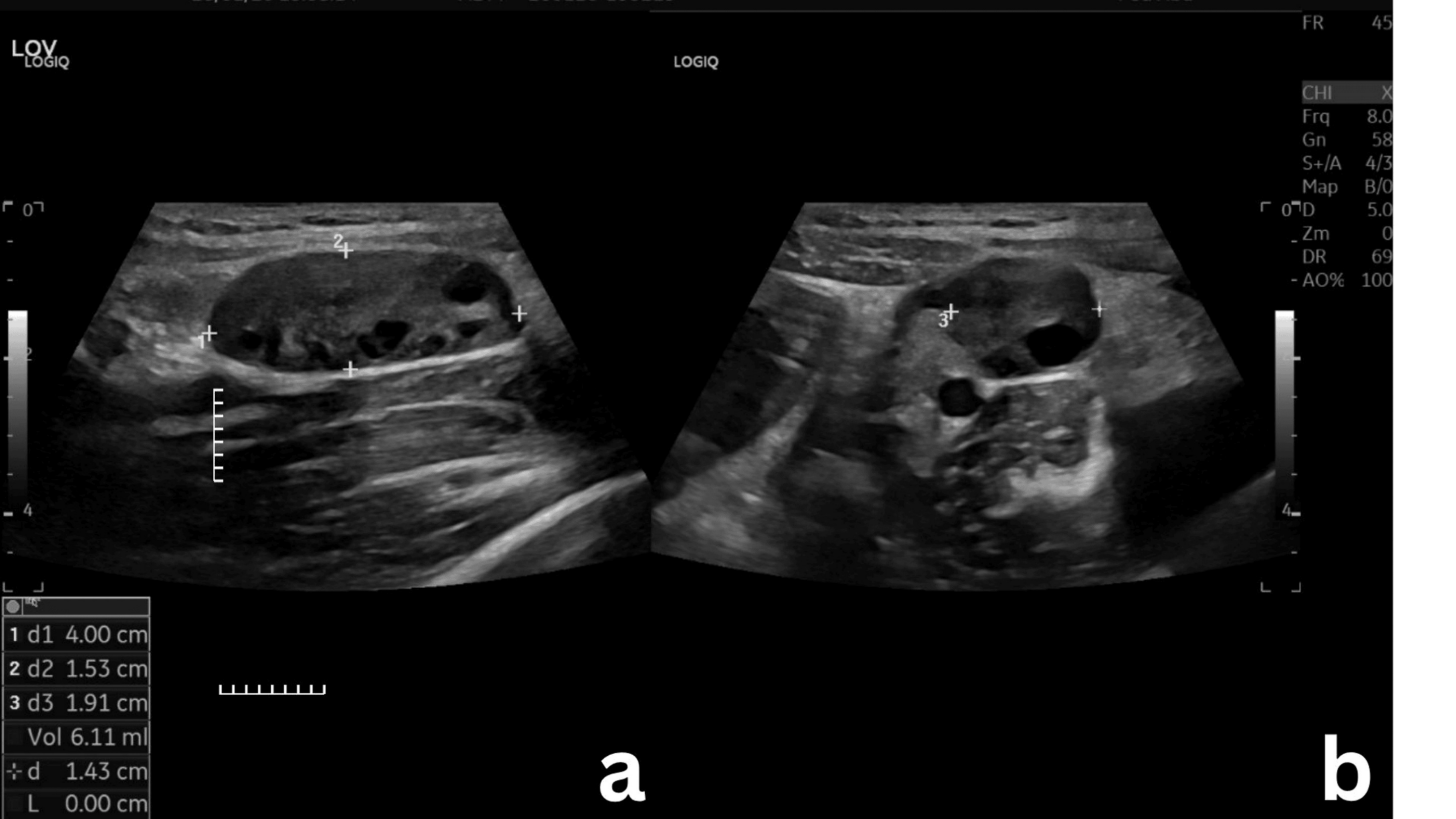
Follow-up ultrasound examination six months after oophoropexy (a) Follow-up ultrasound image demonstrating preserved morphology of the left ovary with normal follicular distribution. (b) Ultrasound measurements confirming decreased ovarian volume (6.11 mL) and normal postoperative appearance.

At the six-month follow-up, ultrasound examination demonstrated a normally positioned left ovary with preserved morphology, normal follicular distribution, and adequate vascularity on Doppler imaging, with an estimated volume of approximately 6 mL and no evidence of recurrent torsion (Figure 5).

## Discussion

Recurrent ovarian torsion in the pediatric population is a rare but clinically significant condition, particularly in cases involving otherwise normal adnexa. Our case is notable for the unusually high frequency of recurrent torsion, with four episodes involving the same ovary over a four-month period despite repeated laparoscopic detorsion and preservation of ovarian viability [3].

Although conservative management with detorsion is currently the standard of care in children and adolescents, it may predispose to recurrence, especially in patients with increased ovarian mobility or underlying anatomic predisposition. Previous studies have reported a higher risk of recurrence in patients with torsion of normal adnexa compared to those with ovarian masses.

Given the repeated episodes in our patient and the substantial risk of further torsion with potential ovarian loss, oophoropexy was considered a necessary step to preserve ovarian function. However, there are no clear guidelines regarding the optimal timing or surgical technique for ovarian fixation.

Various oophoropexy techniques have been described in the literature, including fixation of the ovary to the pelvic sidewall, posterior uterine or abdominal wall, plication or shortening of the utero-ovarian ligament, and fixation to the round ligament of the uterus. Each technique has potential advantages and limitations. Fixation to the pelvic sidewall is widely used and may approximate a more physiological ovarian position; however, it may result in increased tension on the adnexa and possible alteration of tubo-ovarian anatomy. Furthermore, this approach has been associated with postoperative discomfort, particularly during pregnancy, when the ovary would normally ascend into the abdominal cavity [1]. Plication of the utero-ovarian ligament is considered a more physiological approach, as it preserves normal anatomy and may have less impact on future fertility, although it may not be feasible in cases of significant ovarian enlargement. Fixation to the uterosacral ligaments has also been described, but it may be associated with postoperative dyspareunia due to traction on posterior pelvic structures [2]. Fixation to the round ligament has been described as an alternative option, particularly when other techniques are technically challenging or contraindicated, as demonstrated in the present case [2,5]. 

Commonly reported oophoropexy techniques and their characteristics are summarized in Table 1.

**Table 1:** Common oophoropexy techniques and their characteristics Table adapted from Fuchs et al. [1], Yagur et al. [2], and Rajaraman et al. [6].

Technique	Advantages	Limitations
Pelvic sidewall fixation	Technically straightforward; approximates physiological ovarian position	Increased tension on adnexa; possible alteration of tubo-ovarian anatomy; potential discomfort during pregnancy
Utero-ovarian ligament plication	Preserves normal anatomy; potentially better for fertility	Not feasible in enlarged ovaries
Uterosacral ligament fixation	Provides posterior support	Possible dyspareunia due to traction on pelvic structures
Round ligament fixation	Useful alternative when other techniques are not feasible	Limited long-term data; possible alteration of ovarian position
Combined techniques	May provide stronger fixation	Limited evidence; technically more complex

Among these, plication of the utero-ovarian ligament has been proposed by some authors as a preferred approach, as it preserves the normal tubo-ovarian relationship and may have less impact on future fertility [1,2]. However, no comparative studies have definitively established the superiority of one technique over another.

Although oophoropexy is intended to reduce the risk of recurrent torsion, recurrence has been reported following several fixation techniques, including pelvic sidewall fixation, utero-ovarian ligament plication, and round ligament fixation. The available evidence is limited to small case series and case reports, making direct comparison between techniques difficult. Consequently, no single method has demonstrated clear superiority in preventing recurrence, and surgical management should be individualized according to patient characteristics and intraoperative findings.

In addition to these approaches, combined techniques have also been described [6]. For example, simultaneous plication of the ovarian ligament with fixation to the round ligament has been reported as an effective strategy to prevent recurrence in adolescent patients, further highlighting the lack of a single standardized technique [6].

In the present case, the choice of oophoropexy technique was dictated by intraoperative findings. Fixation to the pelvic sidewall was not feasible due to the presence of a markedly dilated pelvic venous plexus, which posed a significant risk of vascular injury. In addition, plication of the utero-ovarian ligament was not technically possible because of the markedly enlarged ovary. Under these circumstances, fixation of the ovary to the round ligament of the uterus represented a practical and anatomically acceptable alternative.

Multiple fixation passes secured with sliding knots were used to distribute tension evenly along the ovary and round ligament, thereby achieving stable fixation while minimizing focal stress on the adnexa.

Concerns have been raised regarding the potential impact of oophoropexy on future fertility, particularly due to the possible disruption of tubo-ovarian anatomy. Nevertheless, available evidence suggests that ovarian function is generally preserved following conservative management and oophoropexy, especially when minimally invasive techniques are employed and excessive tension on the adnexa is avoided [1].

The risk of ovarian loss and its potential implications for future fertility should be carefully discussed with the patient's family. In pediatric patients, preservation of ovarian function is of paramount importance, and counseling should include both the risk of recurrent torsion and the possible consequences of surgical intervention.

A limitation of the present report is the relatively short follow-up period of six months. Although no recurrence or postoperative complications were observed during this interval, longer follow-up is necessary to evaluate the durability of fixation and its potential impact on future reproductive outcomes.

This case highlights the importance of individualized surgical decision-making in pediatric patients with recurrent ovarian torsion. The selection of the oophoropexy technique should be guided by intraoperative anatomy and feasibility, rather than a single standardized approach.

## Conclusions

Ovarian torsion in the pediatric population remains a diagnostic and therapeutic challenge, particularly in cases of recurrence without underlying pathology. This case highlights the importance of early recognition and repeated ovarian preservation through prompt surgical intervention. In patients with recurrent torsion, oophoropexy should be considered to reduce the risk of further episodes and potential ovarian loss. The choice of fixation technique should be individualized based on intraoperative findings and anatomical considerations. Fixation to the round ligament may represent a safe and effective alternative when other techniques are not feasible, without compromising ovarian viability.
